# Feeding behaviour of the nauplii of the marine calanoid copepod *Paracartia grani* Sars: Functional response, prey size spectrum, and effects of the presence of alternative prey

**DOI:** 10.1371/journal.pone.0172902

**Published:** 2017-03-03

**Authors:** Laura K. Helenius, Enric Saiz

**Affiliations:** 1 Department of Environmental Sciences, University of Helsinki, Helsinki, Finland; 2 Tvärminne Zoological Station, University of Helsinki, Hanko, Finland; 3 Institut de Ciències del Mar – CSIC, Barcelona, Catalunya, Spain; University of Connecticut, UNITED STATES

## Abstract

Laboratory feeding experiments were conducted to study the functional response and prey size spectrum of the young naupliar stages of the calanoid copepod *Paracartia grani* Sars. Experiments were conducted on a range of microalgal prey of varying sizes and motility patterns. Significant feeding was found in all prey of a size range of 4.5–19.8 μm, with Holling type III functional responses observed for most prey types. The highest clearance rates occurred when nauplii fed on the dinoflagellate *Heterocapsa* sp. and the diatom *Thalassiosira weissflogii* (respectively, 0.61 and 0.70 mL ind^-1^ d^-1^), suggesting an optimal prey:predator ratio of 0.09. Additional experiments were conducted to examine the effects of the presence of alternative prey (either *Heterocapsa* sp. or *Gymnodinium litoralis*) on the functional response to the haptophyte *Isochrysis galbana*. In the bialgal mixtures, clearance and ingestion rates of *I*. *galbana* along the range of the functional response were significantly reduced as a result of selectivity towards the larger, alternative prey. Paradoxically, relatively large prey trigger a perception response in the nauplii, but most likely such prey cannot be completely ingested and a certain degree of sloppy feeding may occur. Our results are further evidence of the complex prey-specific feeding interactions that are likely to occur in natural assemblages with several available prey types.

## Introduction

Zooplankton grazing is a key process in pelagic ecosystem functioning as a driver and modulator of the vertical particle flux in the water column [1], [2]. Small planktonic copepods are likely the most abundant metazoans on Earth, and have an important role as grazers and as a source of prey for fish and their larvae in coastal as well as pelagic ecosystems (reviewed by Turner [3]). Therefore, aspects of their feeding ecology, including behaviour and the quantification of feeding rates, draw much scientific interest.

Copepod feeding rates can depend on a myriad of factors, such as body mass, temperature, and degree of satiation, as well as food type, quality and concentration [4], [5], [6]. The rate of intake of a particular food type may also be affected by the presence of alternative food choices [7], [8]. Prey switching can take place due to changes in relative food abundance [9], [10], [11], and some evidence has indicated that copepods select particles that are particularly abundant in terms of biomass [12], [13], [14] (but see [15]). Copepods have been shown to discriminate between different types of food and feed selectively, based on size [16], [17] or chemical properties of food types [18], [19]. However, it is not clear to what extent they can distinguish prey quality based on nutritional value alone [20], [21]. For adult copepods, which either generate feeding currents or use active cruising or passive ambush feeding strategies, clearance rates for large particles are higher than those for small particles as a result of the different capture mechanisms involved and the size-dependent probability of prey detection and capture [15], [22], [23]. Concentration-dependent selectivity has been demonstrated in copepods, often indicating higher selectivity with higher food abundance and lower selectivity with food scarcity, but contrasting patterns have also been found [8], [24]. When investigating such consumer-prey interactions in zooplankton, the use of plurialgal experimental mixtures is relevant, because these mirror the complexity of natural ecosystems more closely than studies using a single algal species. Although this multiple prey approach might prove more effective in revealing the underlying mechanisms driving patterns of prey selection and switching thresholds, plurialgal feeding studies using copepods are still rare and may be difficult to interpret (e.g. [8], [10], [17], [25], [26]).

The ecology of the larval stages of copepods is less well known than that of adults. A substantial portion of experimental work on copepod feeding ecology has been conducted on adult or late copepodite individuals, even though nauplii are numerically more abundant and make up a significant part of copepod biomass and secondary production in nature [27], [28]. Moreover, copepod nauplii are major prey of the early stages of fish larvae and can influence fish population dynamics [29]. Because of their ubiquity and their ability to utilize small particles, nauplii may contribute significantly to community grazing rates [30] and constitute a key link between the microbial loop and higher trophic levels [31], [32], [33]. Despite some pioneering work (e.g. [34], [35], [36]), the study of their feeding behaviour and trophic ecology has been frequently neglected until recently [17], [37], [38], [39], [40].

The feeding mechanism of the nauplius larva differs considerably from that of the adult copepod. Although both utilize setae on their respective feeding appendages, food collection by nauplii occurs through movement of the second antennae and mandibles [35], [41], whereas in adults more complex appendages are developed and the second maxilla participates in prey capture [22], [42]. Recent studies confirm that feeding patterns and prey capture of nauplii, long been thought to differ from those of adult copepods and copepodites, are indeed often unique to this early developmental stage [39], [43]. More conflicting, however, are the results on the occurrence of selective feeding in nauplii, because some studies support the idea of selection of large particles [44] whereas others have found no evidence of selective feeding [45].

In this study our main goal was to attain a comprehensive view of feeding behaviour of the nauplius of a marine pelagic calanoid copepod, *Paracartia grani* (formerly *Acartia grani*). To do so, we quantified its functional response on a variety of prey of different sizes and motility patterns. The functional response is a key concept in trophic ecology [46], [47], as it characterizes the capability of a species to exploit resources for survival and further growth and recruitment. The quantitative parameters gained from functional response experiments are paramount as input in models of ecosystem dynamics [48], [49], [50]. Based on the maximum clearance rates exhibited under various prey, we also assessed the prey size spectrum of *P*. *grani* nauplii and determined the optimal prey:predator size ratios. Finally, we also aimed to determine how the simultaneous presence of an alternative prey affects the unialgal functional responses of *P*. *grani* nauplii. Laboratory experiments are typically conducted with single prey suspensions, while in nature copepods are exposed to diverse multispecific prey assemblages. To achieve that goal, we assessed the functional response of *P*. *grani* nauplii to the small algae *Isochrysis galbana* under the presence of an alternative, larger prey for the copepod (the dinoflagellates *Heterocapsa* sp. and *Gymnodinium litoralis*) and quantified the change in the feeding rates as a function of its relative concentration.

## Methods

Feeding experiments were conducted using naupliar stages of the calanoid copepod *P*. *grani*. Nauplii were obtained from the continuous culture at the Institut de Ciències del Mar—CSIC, in Barcelona (Spain), grown in 20 L methacrylate cylinders at 18°C with a 12:12 light:dark cycle and fed with the cryptophyte *Rhodomonas salina*. Cohorts of stage NII nauplii were obtained by the protocol described in detail in Henriksen et al. [37] and summarized as follows: After cleaning the bottom of the copepod culture tanks and allowing the adults to lay eggs for 24 hours, these fresh eggs were siphoned out, counted under the stereomicroscope, and transferred to a new cylinder to hatch and grow in a suspension of *R*. *salina*. The following day, the eggs that were unhatched were removed from the cylinder bottom in order to narrow the body size span of the cohort. Approximately 50 hours after the original egg collection, the nauplii were at the desired developmental stage according to morphological characteristics observed via microscopy (stage NII, Vilela [51]), and were used in the experiments.

The algae used for experiments were the haptophyte *I*. *galbana*, the dinoflagellates *Heterocapsa* sp., *G*. *litoralis* and *Akashiwo sanguinea*, the diatom *Thalassiosira weissflogii*, the cryptophyte *R*. *salina*, and the heterokontophyte *Nannochloropsis oculata*, as well as mixtures of these. All algae used in experiments were grown in batch cultures and diluted daily to keep them in exponential growth. Carbon contents of algae and nauplii were estimated at the Tvärminne Zoological Station (Hanko, Finland) using mass spectrometry (Europa Scientific ANCA-MS 20–20 C/N analyzer) from samples, which had been filtered onto pre-combusted 25mm GF/C filters, dried, and packed into cryovials with vanadium pentoxide. Cell sizes and their carbon contents are summarized in Table 1. Feeding experiments were conducted in 72 mL transparent plastic tissue culture flasks. In the functional response experiments, cell concentration range was 50–150,000 cells mL^-1^ depending on prey species and cell size. Seven concentrations of each suspension were prepared for each prey, with the exception of *N*. *oculata* and *A*. *sanguinea*, for which three concentrations (low, medium, high) were prepared (Table 2). The range of algal concentrations was obtained by successive dilution of stock cultures and the suspensions were adjusted using a Beckman Coulter Multisizer III particle counter fitted with a 100 μm aperture tube, which also provided cell sizes as equivalent spherical diameter (ESD). Three initial flasks, and additionally three control (algae only) and three experimental (algae and nauplii) flasks for each concentration were filled with prey suspension using a multi-step filling procedure to ensure homogeneity between replicates. Nauplii for the experimental flasks were first concentrated into a volume of 250 mL, gently washed several times with filtered seawater to remove previous food particles and added into experimental flasks by volume (ind mL^-1^). The amount of nauplii added to each flask was adjusted according to the algae concentration. Nutrient solution (5 mL f/2 L^-1^) was added to prevent nutrient limitation in the flasks and to compensate for algal growth in the experimental flasks due to naupliar excretion. The control and experimental flasks were sealed with plastic foil to prevent bubble formation, capped, and mounted on a plankton wheel (0.2 rpm), whereas initial flasks were sampled immediately to determine initial prey concentrations. Incubation took place for approximately 24 hours, at 18°C with a 12:12 light:dark cycle. After incubation, nauplii were sieved out from the flasks, preserved using acidic Lugol’s solution (2%), counted and measured. Cell concentrations in the algal suspensions were immediately determined using the Beckman Coulter Multisizer III particle counter. In cases where either cell concentrations were too low or cells were too large for analysis using the particle counter (*G*. *litoralis*, *A*. *sanguinea*), the suspensions were preserved in 1% acidic Lugol’s solution and cell abundance estimated by counting at least 300 cells per sample under an inverted microscope, using either Sedgwick-Rafter counting slides or settling chambers. Due to the very small cell size, *N*. *oculata* concentrations in the experiments were estimated as chlorophyll *a*, determined by measuring the fluorescence, before and after acidification, of 90% acetone extracts of samples filtered onto GF/F filters. Chlorophyll *a* values from the feeding experiments were converted into cell equivalents using a conversion factor obtained from paired chlorophyll *a* measurements and haematocytometer cell counts in the stock cultures.

**Table 1:** Size and carbon and nitrogen contents of *P*. *grani* nauplii and the experimental algae.

Species	Size (±SE, μm)	Carbon content (ng ind^-1^)	Nitrogen content (ng ind^-1^)
*Paracartia grani* nauplii	143 ± 1.3	62	16
*Nannochloropsis oculata*	2.5*	1.6x10^-3^*	n/a
*Isochrysis galbana*	4.5 ± 0.16	7.6x10^-3^	1.1x10^-3^
*Rhodomonas salina*	6.9 ± 0.19	3.8x10^-2^	7.2x10^-3^
*Heterocapsa* sp.	12.1 ± 0.21	2.0x10^-1^	4.3x10^-2^
*Thalassiosira weissflogii*	12.8 ± 0.38	2.1x10^-1^	3.7x10^-2^
*Gymnodinium litoralis*	19.8 ± 0.52	1.35	3.7x10^-1^
*Akashiwo sanguinea*	42.9*	4.6*	n/a

Size is body length for nauplii, and ESD for algae.

*From Saiz et al 2014

**Table 2:** Prey concentrations in the feeding experiments.

Experiment type	Prey species (primary + secondary)	Cell concentration (cells mL^-1^)		Cell biomass (μg C L^-1^)	
		Range	Secondary prey	Range	Secondary prey
Unialgal	*Nannochloropsis oculata*	1x10^4^–1.3x10^5^	-	16–200	-
Unialgal	*Isochrysis galbana*	2300–1.3x10^5^	-	18–920	-
Unialgal	*Rhodomonas salina*	240–1.4x10^4^	-	10–402	-
Unialgal	*Heterocapsa* sp.	135–7500	-	31–1700	-
Unialgal	*Thalassiosira weissflogii*	116–6100	-	29–1500	-
Unialgal	*Gymnodinium litoralis*	112–1700	-	152–2240	-
Unialgal	*Akashiwo sanguinea*	51–556	-	234–2500	-
Bialgal	*I*. *galbana + Heterocapsa* sp.	6800–1.0x10^5^	1600	52–773	323
Bialgal	*I*. *galbana + G*. *litoralis*	6000–8.2x10^4^	295	48–688	398

In the unialgal functional response experiments, the range of initial concentrations is provided. In the bialgal experiments, the primary prey was provided in a range of concentrations, whereas the secondary prey was added at one single intermediate concentration.

Algal mixtures were bialgal combinations of the small sized *Isochrysis galbana*, at five concentrations (derived from the unialgal experimental data), and a fixed concentration of an alternative, larger-sized, prey (either *G*. *litoralis* or *Heterocapsa* sp.). The fixed concentration was determined so that the experiments included cases in which the proportion of carbon was derived evenly as well as disproportionately from both prey species. Bialgal combinations are summarized in Table 2.

Clearance and ingestion rates were determined according to Frost [16]. On two occasions, at the lowest cell concentrations, feeding rates were negative and not statistically significant (t-test, p>0.05) when comparing prey growth rates between the control and experimental flasks [40]. In these two cases, as well as in all the tested concentrations of *N*. *oculata* and *A*. *sanguinea*, feeding rates were deemed negligible (zero). Carbon- and nitrogen-specific ingestion rates and daily rations (% d^-1^) were calculated using the prey and nauplius conversion factors in Table 1. The functional response data were fitted to either Holling type II or III models, using nonlinear least-squares regression, depending on the pattern of clearance rates at low prey concentrations. The appropriate model was chosen by visually inspecting the fitted curves on plotted data, so that curve fits adhered to the data trends as closely as possible and provided reasonable parameters. Additionally, data were initially fitted to both model types, and the goodness of the estimated parameters was assessed by comparing the relative error values of the estimates from both fits, thereby choosing the model with substantially lower relative error. The functions used were as follows:

Holling type II: *I* = (*I*_max_ × *C*) / (*C* + *K*_m_) and *F* = *I*_max_ / (*C* + *K*_m_)
Holling type III: *I* = (*I*_max_ × *C*^2^) / (*C*^2^ + *K*_m_^2^) and *F* = (*I*_max_ × *C*) / (*C*^2^ + *K*_m_^2^)

where *I* and *F* are respectively the ingestion (prey ind^-1^ d^-1^) and the clearance (mL ind^-1^ d^-1^) rates, *I*_max_ is the maximum ingestion rate, *C* is the concentration of prey (prey mL^-1^), and *K*_m_ is the half-saturation constant. Maximum clearance rates (*F*_max_, ±SE) were estimated from ingestion data by non-linear regression, substituting *I*_max_ in the ingestion Holling fits either as *F*_max_ × *K*_m_ (type II) or *F*_max_ × 2*K*_m_ (type III):

Holling type II: *I* = (*F*_max_ × *K*_m_ × *C*) / (*C* + *K*_m_)
Holling type III: *I* = (*F*_max_ × 2*K*_m_ × *C*^2^) / (*C*^2^ + *K*_m_^2^)

This procedure proved to provide *F*_max_ estimates closer to those visually observed on the plots than those obtained from the Holling fits on clearance data. In the case of *Heterocapsa* sp. the *F*_max_ estimate was obtained directly from observed values, since the estimate from the non-linear regression (0.28±0.049SE) proved to be significantly lower than the average of the observed values from the lowest prey concentration in which significant feeding occurred (0.61±0.147SE; t-test, p<0.03).

## Results

### Unialgal experiments

**Fig 1:**
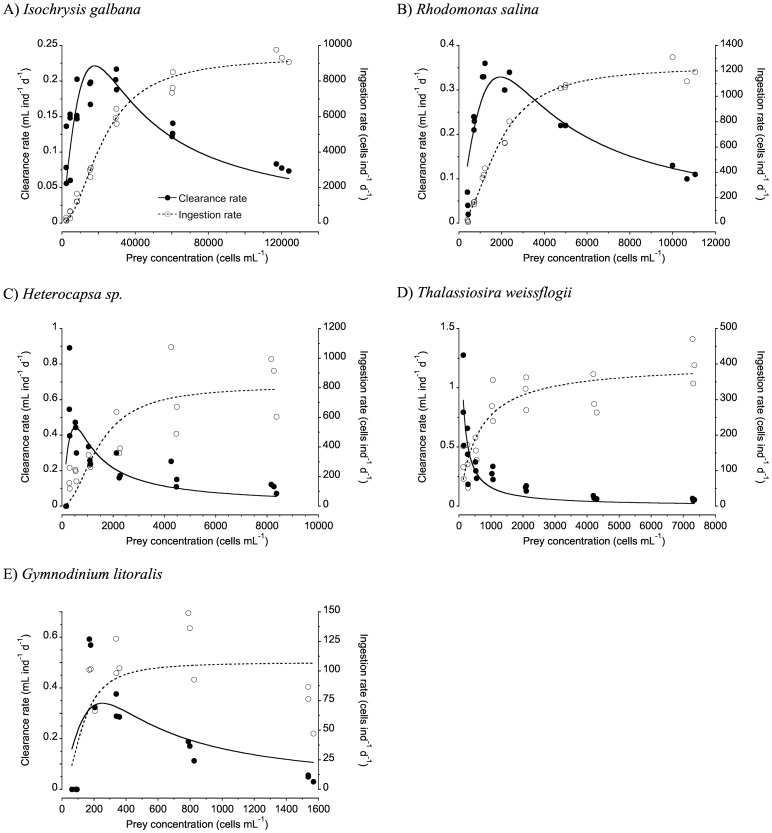
*Paracartia grani* nauplii clearance and ingestion rates as a function of the average prey concentration in the unialgal experiments. Holling type III equations were fitted to data on *Isochrysis galbana*, *Rhodomonas salina*, *Heterocapsa* sp. and *Gymnodinium litoralis*. Holling type II equations were fitted to data on *Thalassiosira weissflogii*.

Significant grazing was found on all prey types except *A*. *sanguinea* and *N*. *oculata*. The feeding behaviour of *P*. *grani* nauplii when offered *I*. *galbana*, *R*. *salina*, *Heterocapsa* sp. and *G*. *litoralis* showed a typical Holling Type III functional response, with a peak in clearance at intermediate prey concentrations and a reduction in foraging effort at lower prey concentrations (Fig 1). When offered *T*. *weissflogii*, however, no evidence of a decline in foraging effort at low prey concentrations was observed and data were fitted to a Holling type II model (Fig 1). Maximum clearance and ingestion rates and the half saturation constant for the prey that elicited significant grazing are shown in Table 3. Maximum clearance rates reached the highest values when feeding on *T*. *weissflogii* and *Heterocapsa* sp., and showed the lowest values for *I*. *galbana*. The ingestion rates increased asymptotically with increasing food concentrations in all experiments, and in terms of cells they reached values up to ca. 10,000 cells per nauplius per day when feeding on *I*. *galbana*, whereas in the case of *G*. *litoralis* the maximum per capita intake was ca. 100 cells per day (Fig 1). When expressed in terms of carbon and nitrogen weight-specific rates (figures not shown), the maximum daily rations by *P*. *grani* nauplii were highest when feeding on the dinoflagellates *G*. *litoralis* (274% body C d^-1^ and 291% body N d^-1^) and *Heterocapsa* sp. (270% body C d^-1^ and 224% body N d^-1^), followed by *T*. *weissflogii* (136% body C d^-1^ and 94% body N d^-1^), *I*. *galbana* (115% body C d^-1^ and 64% body N d^-1^), and finally lowest for *R*. *salina* (74% body C d^-1^ and 53% body N d^-1^).

**Table 3:** Parameters (±SE) of the Holling type II and type III functional response models fitted.

Prey species	*F*_max_ (mL ind^-1^ d^-1^)	*I*_max_ (cells ind^-1^ d^-1^)	*K*_m_ (cells mL^-1^)	R^2^
*Nannochloropsis oculata*	0	0	–	–
*Isochrysis galbana*	0.21±0.007	9401±208.8	22678±1056.4	0.99
*Rhodomonas salina*	0.33±0.012	1234±27.1	1858±84.0	0.99
*Heterocapsa* sp.	0.61±0.147*	813±79.8	1436±317.4	0.76
*Thalassiosira weissflogii*	0.70±0.155	402±30.5	572±157.5	0.77
*Gymnodinium litoralis*	0.42±0.121	107±13.0	130±44.3	0.56
*Akashiwo sanguinea*	0	0	–	–

*F*_max_: maximum clearance rate, *I*_max_: maximum ingestion rate, and *K*_m_: half-saturation food concentration.

*the mean and SE from the actual data at low food concentration are provided instead of the Holling *F*_max_, since the estimates provided by the fit were unrealistically low (see Methods section).

**Fig 2:**
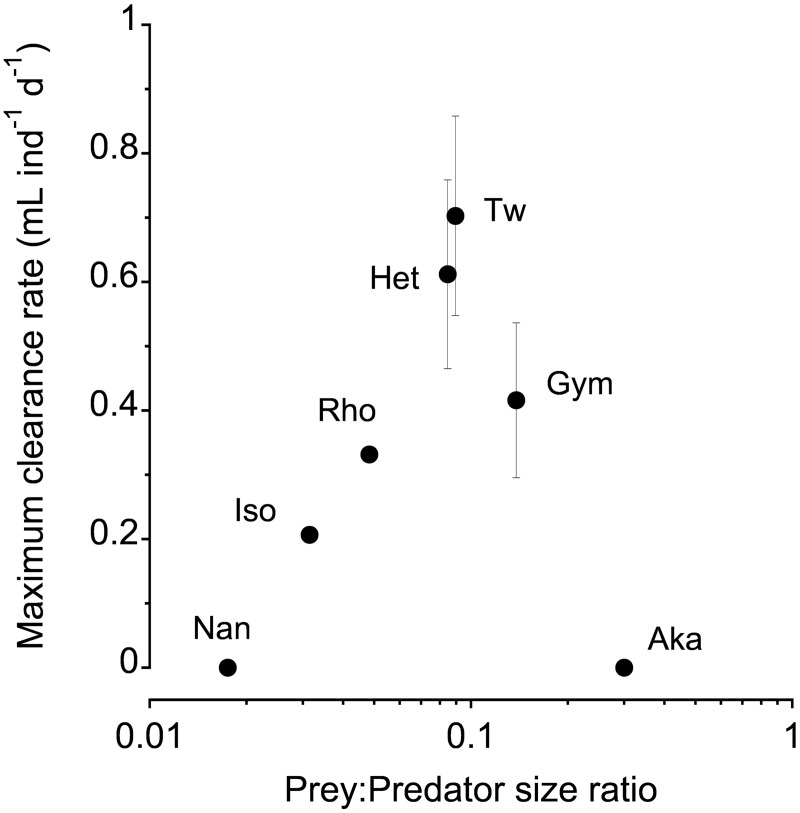
Maximum clearance rates of *Paracartia grani* nauplii in the unialgal experiments as a function of the prey:predator size ratio. Nan: *Nannochloropsis oculata*; Iso: *Isochrysis galbana*; Rho: *Rhodomonas salina*; Het: *Heterocapsa* sp.; Tw: *Thalassiosira weissflogii*; Gym: *Gymnodinium litoralis*; Aka: *Akashiwo sanguinea*

No statistically significant prey ingestion was detected on prey outside of the size range of 4.5–19.8 μm (*N*. *oculata* and *A*. *sanguinea*). The relationship between maximum clearance rates and prey size showed a bell shape, with peak values of 0.6–0.7 mL ind^-1^ d^-1^ when feeding on *T*. *weissflogii* and *Heterocapsa* sp., and a decline in performance at smaller and larger prey sizes (Fig 2).

### Bialgal experiments

**Fig 3:**
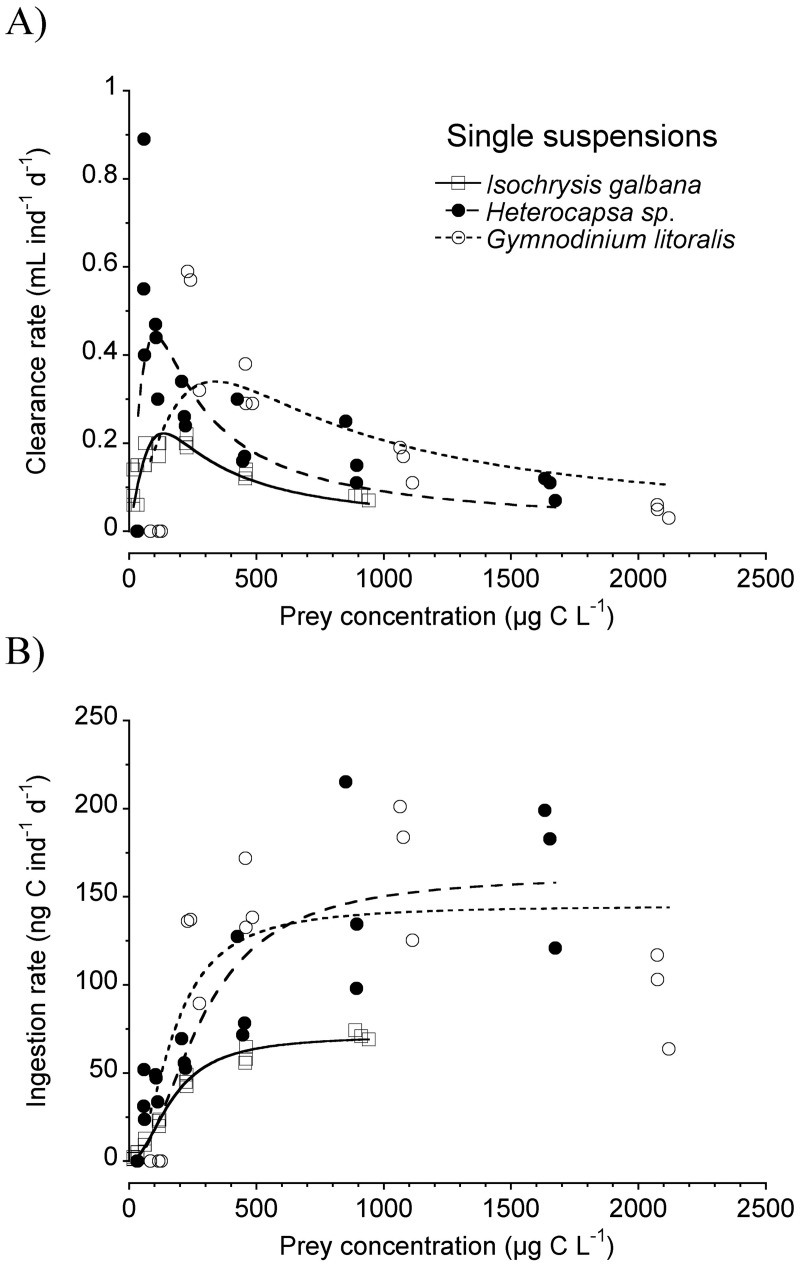
Carbon feeding rates of *Paracartia grani* nauplii on unialgal suspension of the three algae used in the mixture experiments. Clearance (A) and ingestion (B) rates as a function of the average prey concentration. Fits like in Fig 1.

For the sake of the analysis, Fig 3 shows the single-prey carbon-based functional responses of the three prey items used in the bialgal combinations. Clearance rates peaked at higher concentrations (around 300 μg C L^-1^) in the case of *G*. *litoralis*, while for the other two prey peaks occurred at much lower prey concentrations (ca. 100–150 μg C L^-1^) (Fig 3). Maximum carbon ingestion rates were similar for *Heterocapsa* sp. and *G*. *litoralis* (ca. 150 ng C ind^-1^ d^-1^), whereas for *I*. *galbana* maximum ingestion rate values were halved (Fig 3). Satiation occurred at concentrations above ca. 600 μg C L^-1^.

**Fig 4:**
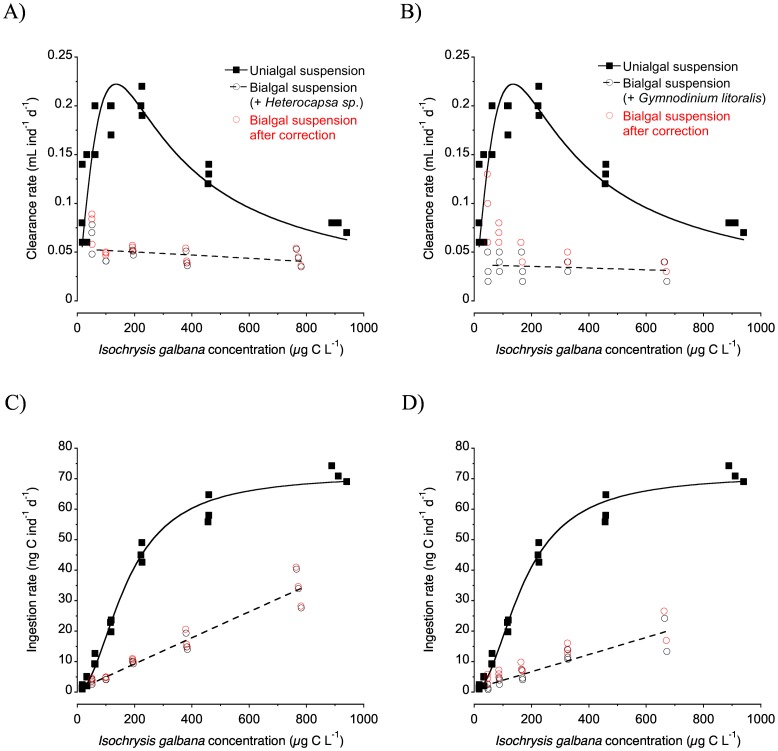
Effects of the presence of alternative prey on the functional response of *Paracartia grani* nauplii on *Isochrysis galbana*. (A) Clearance rate as a function of *Isochrysis galbana* concentration in unialgal suspension and in mixture with *Heterocapsa* sp. (B) Clearance rate as a function of *Isochrysis galbana* concentration in unialgal suspension and in mixture with *Gymnodinium litoralis*. (C) Ingestion rate as a function of *Isochrysis galbana* concentration in unialgal suspension and in mixture with *Heterocapsa* sp. (D) Ingestion rate as a function of *Isochrysis galbana* concentration in unialgal suspension and in mixture with *Gymnodinium litoralis*. Unialgal data were fit to non-linear equations like in Fig 1. For the bialgal experiments, correction for *Isochrysis*-sized particles generated when feeding on the larger prey are also shown (see text). Mixed suspension data were fit with linear equations (only shown for the uncorrected values).

Under the presence of an alternative prey, clearance rates on *Isochrysis galbana* were severely depressed (Fig 4). Instead of a Holling type III shape, clearance rates were flattened, with no significant correlation with *I*. *galbana* concentration in either of the two experiments (p>0.17 in both cases; Fig 4A and 4C). Clearance rates on *I*. *galbana* under the presence of the alternative prey *Heterocapsa* sp. and *G*. *litoralis* averaged 0.048±0.0031SE and 0.035±0.0031SE mL ind^-1^ d^-1^, respectively; when both were compared, clearance rates were significantly lower in the case of the mixture suspension with *G*. *litoralis* (two-tailed t-test, p<0.004). The ingestion rates of *I*. *galbana* in the mixtures were much lower than in the unialgal suspension, and increased linearly with the concentration of *I*. *galbana* (p<0.001, in both cases; Fig 4B and 4D). The rate of increase was significantly lower when the alternative prey was *G*. *litoralis* (covariance analysis using food concentration as covariate, p<0.006; Fig 4B and 4D).

Regarding the secondary algae in the mixture suspensions, clearance and ingestion rates on *Heterocapsa* sp. in the bialgal suspensions averaged 0.12±0.015SE mL ind^-1^ d^-1^ and 37.7±4.49SE ng C ind^-1^ d^-1^, respectively, and were not correlated with the abundance of *I*. *galbana* in the incubations (Table 4; p>0.16 in both cases). A Tukey HSD test after ANOVA, however, showed that clearance and ingestion rates on *Heterocapsa* sp. were both ca. 63% lower at the lowest *I*. *galbana* concentration tested in the bialgal experiments (Table 4; p<0.05; one outlier removed). Clearance and ingestion rates on *G*. *litoralis* in the bialgal suspensions averaged 0.63±0.051SE mL ind^-1^ d^-1^ and 178±9.4SE ng C ind^-1^ d^-1^, respectively, and were not correlated with the abundance of *I*. *galbana* in the incubations (Table 4; p>0.5 in both cases). Clearance and ingestion rates on *G*. *litoralis* as the secondary alga did not differ among *I*. *galbana* concentrations (Table 4; ANOVA test, p>0.25). Overall, the feeding rates observed for the secondary prey in the bialgal experiments diverged from the values obtained in single suspension (Table 4), with lower rates in the case of *Heterocapsa* sp. and higher rates on the case of *G*. *litoralis* (Table 4).

**Table 4:** Feeding rates on the alternative prey in the bialgal experiments.

Alternative prey	*Isochrysis galbana* average concentration (±SE, μg C L^-1^)	Alternative prey average concentration (±SE, μg C L^-1^)	Clearance rate on alternative prey (±SE, mL ind^-1^ d^-1^)	Ingestion rate on alternative prey (±SE, ng C ind^-1^ d^-1^)
*Heterocapsa sp*.	–	*328±50*.*7*	*0*.*24±0*.*028*	*76±11*.*1*
*Heterocapsa sp*.	52±0.2	347±2.8	0.04±0.017	13±5.8
*Heterocapsa sp*.	100±0.2	296±1.3	0.12±0.017	35±4.8
*Heterocapsa sp*.	194±0.8	331±5.3	0.13±0.012	43±3.3
*Heterocapsa sp*.	382±1.4	324±9.5	0.18±0.038	58±10.3
*Heterocapsa sp*.	773±4.8	316±3.3	0.12±0.007	39±1.8
*Gymnodinium litoralis*	–	*358±49*.*2*	*0*.*41±0*.*057*	*134±10*.*7*
*Gymnodinium litoralis*	48±0.2	238±15.5	0.83±0.174	192±31.3
*Gymnodinium litoralis*	88±0.1	317±9.4	0.51±0.043	161±11.8
*Gymnodinium litoralis*	168±1.3	285±13.4	0.64±0.033	182±10.6
*Gymnodinium litoralis*	327±0.1	306±11.1	0.52±0.055	157±12.0
*Gymnodinium litoralis*	668±4.0	332±24.6	0.64±0.172	207±41.5

Average concentration of the primary prey, *Isochrysis galbana*, is also provided. Feeding rates on the alternative prey in unialgal suspensions at similar concentrations (see Fig 3) are also provided for comparison (in italics).

Fernández [34] and Frost [52] both pointed out the fact that in copepod feeding experiments dealing with algal mixtures using automatic particle counters like the Coulter counter, the generation of small-sized detritus by copepod feeding may overlap with the size range of the smallest prey in the mixtures, resulting in underestimating ingestion of the latter. Using the Coulter data corresponding to the experiments of *P*. *grani* nauplii fed on unialgal suspensions of *Heterocapsa* sp. and *G*. *litoralis*, we quantified the statistically significant amount of particles produced (compared to the control flasks without predators) that fell within the size range of *I*. *galbana*. In the case of *Heterocapsa* sp. we found a linear relationship between *Heterocapsa* sp. consumption and the increase in *Isochrysis*-sized particles (S1 Fig); when fed *G*. *litoralis* no clear linear trend could be defined and on average 2.8 small particles generated per each *G*. *litoralis* cell consumed. Clearance and ingestion rates of *I*. *galbana* in the bialgal experiments were then recalculated taking into account the production of *Isochrysis*-sized particles when feeding on the larger prey types. Overall the corrected data, also shown in Fig 4, confirms the absence of substantial flaw on our experimentally determined feeding rates.

**Fig 5:**
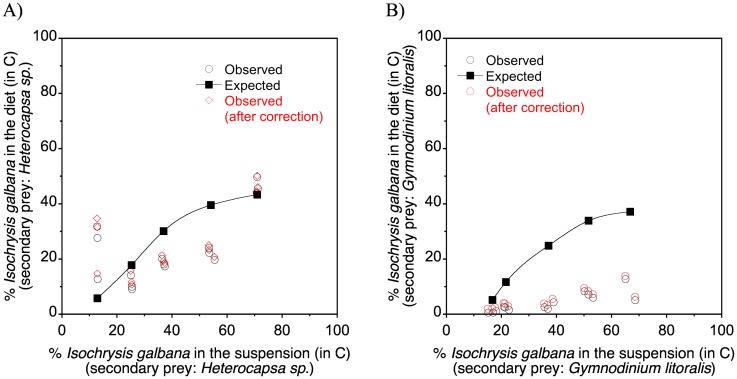
Observed and expected relative contribution of the primary and secondary prey in the diet of nauplii as a function of their relative carbon contribution in the bialgal suspension. (A) *Isochrysis galbana* in the bialgal suspension composed of *Isochrysis galbana* and *Heterocapsa sp*.; (B) *Isochrysis galbana* in the bialgal suspension composed of *Isochrysis galbana* and *Gymnodinium litoralis*. Corrections for *Isochrysis*-sized particles generated when feeding on the larger prey are also shown (see text). Line is spline fit to the data.

Fig 5 shows the prey contribution in terms of carbon to the diet of *P*. *grani* nauplii as a function of its relative contribution in the bialgal suspensions. We also calculated the expected prey contribution to the diet, given a certain relative prey concentration, using the data from the single suspension functional responses and assuming no change in nauplii behaviour between uni- and bialgal experiments. In the case of the primary prey, *I*. *galbana*, the observed contribution to the diet over a range of food concentrations was overall lower than expected (Fig 5). This effect was much more intense when *G*. *litoralis* was present as alternative prey, whereas in the case of *Heterocapsa* sp. as alternative prey it was only evident at the intermediate range of concentrations; in fact, at the lowest bialgal food suspension *I*. *galbana* contributed more to diet than expected (Fig 5). In neither case did the inclusion of the mentioned by-produced *Isochrysis*-sized particles result in substantial changes in the nauplius feeding rates (Fig 5). Due to the substantial decrease in the intake of *I*. *galbana*, the relative contribution of the secondary prey was in most cases higher than expected, in spite of the fact that the intake of *Heterocapsa* sp. in the bialgal suspension was also decreased when compared with the unialgal suspension (Table 4).

In the bialgal experiments, the total amount of food available ranged from ca. 300 to 1100 μg C L^-1^. When *Heterocapsa* sp. was the secondary prey there was a significant correlation between the total amount of food available in the bialgal suspension and the total food intake by the nauplii (r = 0.71, p<0.004; Fig 5). No significant correlation between these two variables was found when the secondary prey was *Gymnodinium litoralis* (p>0.34; Fig 5).

## Discussion

### Unialgal experiments: Feeding functional response

Given the limited existing literature on nauplius feeding, the feeding rates and subsequent daily rations found in our experiments were overall comparable to those reported for nauplii of small or medium-sized copepod species. Henriksen et al. [37] reported maximum values of clearance rate and daily ration in the order of, respectively, 0.3–0.4 mL ind^-1^ d^-1^ and ca. 250% body C d^-1^ when fed on *Heterocapsa* sp., and of, respectively, 0.4–0.5 mL ind^-1^ d^-1^ and ca. 150% body C d^-1^ when fed on *T*. *weissflogii*, for *P*. *grani* nauplii. Berggreen et al. [36] reported clearance rates up to 0.2–0.4 for similarly sized nauplii of *A*. *tonsa*, but food concentration in their experiments may not have been low enough to trigger maximum rates (0.6–0.7 ppm, equivalent to e.g. ca. 180 μg C L^-1^ of *Rhodomonas baltica* or ca. 300 μg C L^-1^ of *I*. *galbana*). The feeding rates we obtained for *P*. *grani* nauplii are also in agreement with maximum clearance rates reported for the larger nauplii of *Eurytemora affinis* and *A*. *tonsa* on natural seston (<1 mL ind^-1^ d-1) [45]. For the ambush cyclopoid copepod *Oithona davisae*, Henriksen et al. [37] and Almeda et al. [38] reported maximum clearance rates of 0.3 and 0.4 mL ind^-1^ d^-1^ for NII-NIII nauplii fed on *Heterocapsa* sp. and *Oxyrrhis marina* respectively, in agreement with the range of values reported for the same species under a broad assortment of prey types by Saiz et al. [40]. In all these studies, given the low motility and ambush behaviour of *O*. *davisae* nauplii [37], maximum daily rations were lower than for similarly sized calanoid nauplii and did not exceed 120% body C d^-1^.

The shape and magnitude of the functional response contains information on the capability of a certain species to cope with environmental food conditions. Additionally, the sigmoidal or Holling type III response, in which there is a threshold food concentration below which clearance rates decline, may act at the prey community level as a stabilizing mechanism [11], [53]. In our experiments using *P*. *grani* nauplii, most of the single prey functional responses followed a Holling type III model. Hence, type III responses were found when *P*. *grani* nauplii were fed on the smallest cells *I*. *galbana* and *R*. *salina*, which may provide a lower (per capita) energetic gain at low prey concentrations (*I*. *galbana*: threshold below 18000 cells mL^-1^ or 137 μg C L^-1^; *R*. *salina*: threshold below 1900 cells mL^-1^ or 72 μg C L^-1^) but also when offered the large dinoflagellate *G*. *litoralis* (threshold below ca. 250 cells mL^-1^ or 338 μg C L^-1^). No such feeding threshold was observed when the prey was the non-motile diatom *T*. *weissflogii*, even when its concentration in our experiments was as low as 140 cells mL^-1^ or 28 μg C L^-1^. Surprisingly, a type III functional response was indeed observed when the similarly sized dinoflagellate *Heterocapsa* sp. was offered (with a threshold below 300 cells mL^-1^ or 60 μg C L^-1^).

At low prey concentrations, the energetic gain from prey intake may fail to compensate for the costs of searching for and collecting the prey [5], [54]. In copepods, this trade-off can reflect in changes in the relative contribution of their behavioural components and in the outcome of a sigmoidal or type III response [55]. Sarnelle and Wilson [53] recently suggested that type III responses are more common than previously thought in zooplankton, and have not been taken into consideration because of scarce observations at low food levels. Thus, it could be argued that when a type II model is found it could merely be the consequence of not having reached prey concentrations low enough to trigger the type III behavioural pattern. Yet in our experiments at similar low cell concentrations of *Heterocapsa* sp. a type III response was evident.

We do not fully understand why *P*. *grani* nauplii should exhibit a type II response when fed on the diatom instead of the type III response observed with the other prey. It could be a mere consequence of variability among replicates at the very low food concentrations. However, the same discrepancy was found in the study of Henriksen et al. [37], conducted with nauplii of the same copepod species, *P*. *grani*, which were also offered *T*. *weissflogii* and *Heterocapsa* sp. If the data in their Figs 1 and 2 are observed carefully, a type III model when fed on *Heterocapsa* sp. and a type II model when fed on *T*. *weissflogii* can be discerned, confirming the pattern found in the current study. Reports on the functional response of *Acartia tonsa* also show occasional discrepancy in functional response models depending on prey type [56], [57].

The motility of the prey has implications on its detection by the predator, but also on encounter rate, by increasing the relative speed between predator and prey [58]. As *P*. *grani* nauplii are active swimmers [37], the motility of the dinoflagellate *Heterocapsa* sp. does not contribute substantially to the predator-prey encounter rate, given the difference in swimming speed between predator and algal prey [37], [58]. Remote detection of motile prey has been observed in nauplii of the related species *A*. *tonsa* [39], but for non-motile prey like *T*. *weissflogii* the mechanisms of remote detection by nauplii are unknown and may depend on chemical cues. Recent assessments, however, oppose the possibility of remote chemically mediated sensing (olfaction) in copepods, and advocate for mechanoreception as the major underlying mechanism, even for nonmotile prey, while chemical cues might be more important for assessing palatability [22], [59], [60]. In this regard, Henriksen et al. [37] observed that although *P*. *grani* attack distances to *T*. *weissflogii* and *Heterocapsa* sp. did not differ, handling times on the former were longer (albeit not significantly). Moreover, in 36% of the attacks on *T*. *weissflogii* the prey was rejected, perhaps due to the presence of thecae in the diatom, which may impede its intake by the nauplii [37]. We can speculate that for a similar prey intake these differences in rejection rate may favour higher clearance rates on the diatom, particularly at very low prey concentration, therefore resulting in a type II response. Other characteristics of the prey (e.g. differences in exudate quality or production rate [61]) may also tentatively help to explain a comparatively higher degree of feeding activity at very low prey concentrations [62].

### Unialgal experiments: Prey size spectrum

The magnitude of the predator-prey flow of matter in pelagic systems is bound by the predator's prey size spectrum, which constrains the amount of interactions and the degree of overlap between trophic levels, and therefore defines the structural properties of the food web. The prey size spectra of planktonic predators are commonly bell-shaped, with an optimum, and typically expand into larger sizes through ontogeny [36], [63], [64]. Several factors are involved in shaping it (motility, escape ability, quality, toxicity, palatability, etc.), but usually body size has a major role in the probability of a potential prey to be eaten. The positive association between prey size and clearance rate is maintained until a size threshold, where prey handling and intake becomes difficult or prey escape ability increases (e.g. [40]). Planktonic prey:predator size ratios are dependent on the characteristics of each planktonic functional group and often fall far from the typically 0.1 ratio assumed in the ecological literature [36], [63], [65]. Berggreen et al. [36] reported prey:predator size ratios of 0.04–0.05 as optimal for the closely related *A*. *tonsa* nauplii (NII to NVI). In our study, however, the nauplii of *P*. *grani* had an optimum (highest *F*_max_ values) at a higher prey:predator size ratio (0.09). Our prey:predator size ratios are difficult to compare with others reported in the literature, as the few available are usually not obtained from true *F*_max_ values conducted at very low prey densities (e.g. [36]). The work of Fernández [34], [35] on *Calanus pacificus* nauplii, of a much larger body size (ca. 300–500 μm total body length), provided actual functional response data for a few phytoplankton prey. With the data available in Fernández [34], [35], maximum clearance rates of *C*. *pacificus* NIII were achieved at prey:predator size ratios of 0.04, whereas from NIV onwards maximum clearance rates peaked at prey:predator size ratios of 0.09. These values are overall similar to the ones reported here, and lower than the optimal relative prey size of 0.14 for early nauplii of the cyclopoid copepod *O*. *davisae* reported by Saiz et al. [40]. The rather high relative prey size of the *O*. *davisae* nauplii was attributed by Saiz et al. [40] to the strict ambush behaviour of cyclopoid copepods, which requires prey that are large and motile enough to both generate a mechanical cue strong enough to be perceived, and increase the prey-predator relative velocity and therefore enhance encounter rates [58]. However, we cannot rule out that some of the differences in the values mentioned above are likely dependent on the range and variety of prey used in the different studies, which are commonly quite limited.

Adult copepods are generally inefficient at grazing particles of a size less than 5–10 μm [36], [65]. According to our results, feeding on very small prey by *P*. *grani* nauplii also proved inefficient or negligible, indicated here by the low clearance rates on *I*. *galbana* and the lack of detectable ingestion on the smaller *N*. *oculata* (ESD 2.5 μm). Conceivably, markedly small prey may either not be perceived or not be captured. In fact, the nauplii of *A*. *tonsa*, which are rather active hop-and-sink swimmers similar to the nauplii of the related *P*. *grani* [37], [66] do not generate a true feeding current but engage in raptorial feeding behaviour and capture cells individually [39]. Similar lack of efficient feeding on very small prey (<2 μm) has been reported for nauplii of other species [34], [36], [67].

In our study the two most optimal prey types, determined by the highest clearance rates, appeared to be the dinoflagellate *Heterocapsa* sp. and the diatom *T*. *weissflogii* (Fig 2), which obviously differ in motility. Both the nauplii of *A*. *tonsa* and *P*. *grani* can feed on non-motile prey [36], [37], but the underlying mechanisms of prey detection have not been described yet [39]. As mentioned before, the lack of motility of *T*. *weissflogii* may not substantially affect its encounter rate with *P*. *grani* nauplii, since the contribution of the prey velocity component to encounter is likely to be rather low, and for this reason maximum clearance rates on both prey were similar. Regarding ingestion rates, and similarly to Henriksen et al. [37], in our experiments maximum cell ingestion rates of *P*. *grani* nauplii feeding on *Heterocapsa* sp. were considerably higher than those on *T*. *weissflogii*. Palatability and other qualities could contribute to the lower ingestion rates on *T*. *weissflogii*. Henriksen et al. [37] reported observations of prey rejection, broken cells and relatively longer handling times when *P*. *grani* nauplii were fed on *T*. *weissflogii*. Moreover, silicon frustules presumably render the diatom a more substantial prey, which fills the nauplius gut faster and therefore limits ingestion rate [37].

As prey size increases, the probability of capture and ingestion decreases. Larger prey may escape better and may require longer handling times. However, handling times in copepods are usually very short and may not markedly limit ingestion rates [22], [37], [39]. Another constraint in the upper bound of the prey size spectrum is mouth size, which may prevent full prey body intake as reported by Saiz et al. [40]. Given the size of the *P*. *grani* nauplii we used (143 μm), according to the anatomical works of Fanta [68], [69] on similarly-sized nauplii (NIII of *Euterpina acutifrons*: 141 μm body length, mouth size: ca. 14.1 μm × 6.9 μm; NIV of *Oithona ovalis*: 135 μm body length, mouth size: ca. 13.5 μm × 1.2 μm) the mouth of *P*. *grani* nauplii is expected to be rather small and presumably would result in a certain degree of sloppy feeding on prey much larger than *R*. *salina*. This fact may explain the apparently high daily rations obtained in our experiments when nauplii were fed on relatively large prey (i.e. *G*. *litoralis*, *Heterocapsa* sp.), as well as other extreme values found in the literature [37], [40], [70]. As Møller [71] found in adult copepods, when a prey type is comparatively large for a predator, the outcome of prey removal incubation experiments may overestimate the realized ingestion rate to a certain extent, as a significant part of the prey carbon is lost as dissolved organic carbon. Given the ecological implications of this process, further studies directly quantifying the extent and relevance of sloppy feeding are required.

### Bialgal experiments: Effects of alternative prey on feeding functional response

**Fig 6:**
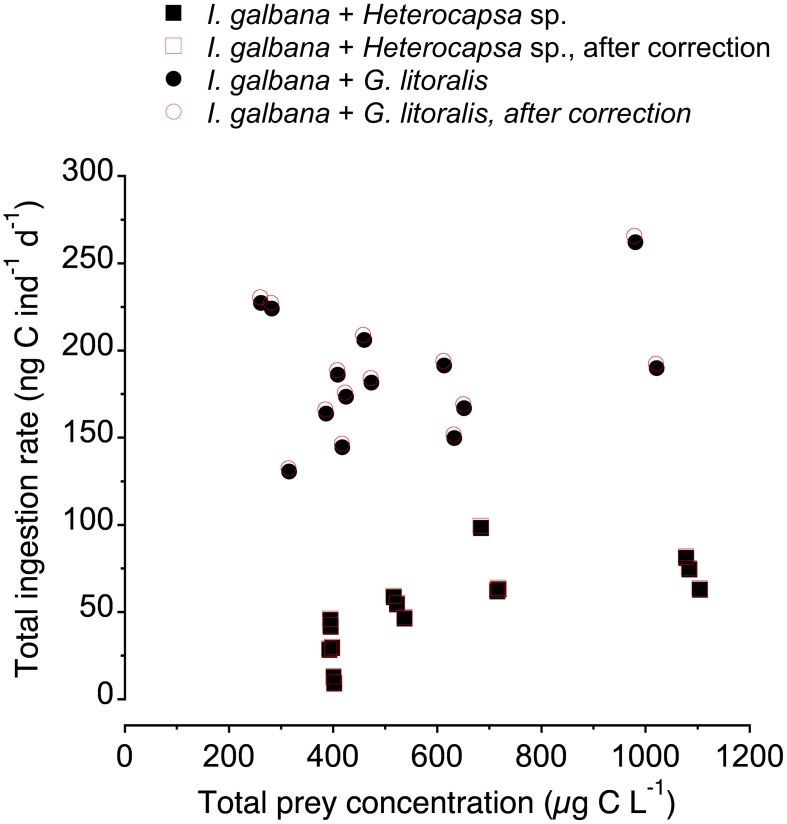
Total (both prey) ingestion rate of *Paracartia grani* nauplii as a function of total prey concentration in the bialgal experiments.

Copepods in their natural environment experience a diverse assemblage of organisms as potential prey, with different sizes and shapes, patterns of motility, and elemental and biochemical compositions. Mimicking this diversity in the laboratory often proves unfeasible; hence most experiments on copepod feeding conducted in the laboratory have used unialgal suspensions. In the few cases with multiple prey assemblages, prey types are commonly offered in fixed proportions and do not take into account the intrinsic differences in the functional relationship between prey concentration and clearance rate among the differently sized prey (e.g. [44], [52]). In our study, we opted for a different approach. We first studied, in single suspension, the feeding functional response of the prey to be used in the bialgal experiments, and then we assessed the effect of the presence of the secondary prey on the functional response towards the primary one (*Isochrysis galbana*). The design of our bialgal experiments resulted in different relative amounts of both offered prey along the range of prepared bialgal suspensions (13–71% of carbon contributed by *I*. *galbana* when *Heterocapsa* sp. was the alternative prey; 17–67% of carbon contributed by *I*. *galbana* when *G*. *litoralis* was the alternative prey). In single suspension threshold limiting carbon concentrations were ca. 500–600 μg C L^-1^ (Fig 3B), hence the changes in foraging behaviour due to the presence of alternative prey in our experiments encompassed food limiting and food satiating conditions (Fig 6).

The functional response of *P*. *grani* nauplii on *I*. *galbana* was notably affected by the presence of alternative prey. Both the maximum clearance and ingestion rates were substantially lower, and the shape of the functional response turned into a non-satiated (linear) relationship instead of the Holling type III model displayed under the single prey suspension.

The potential bias result of the generation of small-sized particles falling into the size range of *I*. *galbana* due to the feeding activity on the larger prey proved not to be relevant and did not affect the above-mentioned conclusions. Overall, in terms of clearance and ingestion rates the feeding rates on *I*. *galbana* were clearly suppressed when in bialgal suspension with respect to the ones observed in unialgal suspension. The detriment on clearance and ingestion rates of *I*. *galbana* was more pronounced when the alternative prey was *G*. *litoralis* rather than *Heterocapsa* sp. This could be a consequence of the larger size of *G*. *litoralis* that may result in longer prey handling time or even differences in digestion time, or alternatively could be related to the enhanced feeding on *G*. *litoralis* under the presence of *I*. *galbana* (Table 4).

We have seen that the presence of an alternative, larger prey at a constant concentration had a negative effect on the feeding on *I*. *galbana*. It seems that the higher clearance rates for the comparatively larger prey (Fig 2) override the effect of food concentration and relative proportion of each prey. Thus, in the plots shown in Fig 5 in most cases the mixture data falls below the expected daily rations, indicating that when the alternative, larger prey was present, *P*. *grani* nauplii substantially reduced foraging effort on *I*. *galbana* in relation to its relative abundance (except at the two extremes of the range of concentrations tested using *Heterocapsa* sp. as the alternative prey). Similarly, Fernández [34] and Smith et al. [72] reported for nauplii of the copepods *C*. *pacificus* and *A*. *tonsa*, respectively, a positive selection for larger prey when the nauplii were offered algal mixtures. Adult copepods in general also exhibit a similar pattern of selection of the larger prey over the small one [17], [52].

Our study also provides evidence of changes in the feeding behaviour of the nauplii when offered prey mixtures. The outcome of feeding experiment with mixtures is not the result of an additive process taking into account the unialgal functional responses. Moreover, the behaviour of the nauplii towards the alternative prey was also affected by the presence of *Isochrysis galbana* in the mixture. In the case of *Heterocapsa* sp. as alternative prey, the ingestion rates on *Heterocapsa* sp. were lower than expected (in single suspension), as could be anticipated given the occurrence of *I*. *galbana*, which was also consumed at the same time. Surprisingly, *G*. *litoralis* in mixtures was ingested at higher rates than expected (i.e. in single suspension). Unfortunately, we have no clear explanation for the underlying mechanisms responsible for that enhancement. We must note, however, the paradox between the fact that the larger prey appear to be eaten preferentially with respect to *I*. *galbana*, likely because of a better perception of them and higher clearance rates, and at the same time feeding on those large prey certainly must involve a degree of sloppy feeding, i.e. not full consumption of the prey, due to the comparatively large size given the size of the nauplii (and presumably their mouth, see discussion section on prey size spectrum). We believe that the feeding rates on the larger prey, estimated from removal experiments in bottles, may not fully represent the actual prey biomass intake by the nauplii, particularly in the case of *G*. *litoralis* (42.9 μm ESD). This fact may help explain the increased removal of *G*. *litoralis* in the bottles, not necessarily accompanied by an increase of similar magnitude in the actual intake of the prey biomass. In the case of *Heterocapsa* sp. as the alternative prey of a much smaller size (12.1 μm ESD), this effect would be less relevant.

## Conclusions

Our study investigated the feeding behaviour of the nauplius of the calanoid copepod *P*. *grani*, taxonomically pertaining to a copepod family frequently found in estuaries and coastal waters around the world. Feeding functional responses with different prey types typically showed a peak in clearance rate at intermediate food concentrations and a sigmoidal ingestion response, distinctive of Holling type III responses. The prey size spectrum was bell-shaped and constrained within the prey size range ca. 4–20 μm, with a peak in maximum clearance rate at prey:predator size ratios of 0.09. It is conceivable, however, that most likely the tested prey in the upper bound of the spectrum cannot be efficiently ingested due to excessive size and may result in sloppy feeding.

The feeding functional responses in mixtures of differently-sized prey differ in shape and magnitude from those exhibited in single prey suspension. When in mixtures, copepod nauplii select positively for the larger prey, affecting the functional response of the smaller prey. Our results have showed that feeding behaviour is not additive, and ingestion rate and clearance rate values obtained from single suspension experiments may not be directly applicable to predict values in circumstances with several prey types. Further experimental studies coupled with high-speed video observations are necessary to improve our comprehension of the underlying mechanisms driving feeding rates in nature, where copepods are exposed to prey assemblages of multiple attributes and concentrations.

## Supporting information

S1 Fig Abundance of *Isochrysis galbana*-sized particles generated while feeding in unialgal suspensions of either *Heterocapsa* sp. or *Gymnodinium litoralis*. (TIFF)
